# Pathology in the legal framework of European and German medical device law: Operation, use and in-house manufacture of in vitro diagnostic medical devices

**DOI:** 10.3205/000335

**Published:** 2024-10-11

**Authors:** Andy Kahles, Hannah Goldschmid, Anna-Lena Volckmar, Daniel Kazdal, Ulrich M. Gassner, Michael Vogeser, Monika Brüggemann, Karl-Friedrich Bürrig, Vanessa Kääb-Sanyal, Christa Flechtenmacher, Peter Schirmacher, Albrecht Stenzinger

**Affiliations:** 1 Institute of Pathology, Heidelberg University Hospital, Heidelberg, Germany; 2 Faculty of Law, Augsburg University, Augsburg, Germany; 3 Institute of Laboratory Medicine, LMU University Hospital, LMU Munich, Germany; 4 Clinic for Internal Medicine II, Section for Special Hematology Diagnostics, University Hospital Schleswig-Holstein, Kiel, Germany; 5 Professional Association of German Pathologists, registered association, Berlin, Germany

**Keywords:** IVDR, quality management, legislation, regulatory requirements, laws and regulations, in vitro diagnostic medical devices (IVD)

## Abstract

Institutes for pathology act as operators, users and in-house manufacturers of in vitro diagnostic medical devices and are subject to national and European regulations depending on their function. The entry into force of the EU regulation on medical devices (Regulation (EU) 2017/745, MDR) and the EU regulation on in vitro diagnostic medical devices (Regulation (EU) 2017/746, IVDR) resulted in a need for regulatory adjustments to German medical device law. This has created a new legal framework in which institutes for pathology operate, depending on their function as users, operators or in-house manufacturers of in vitro diagnostic medical devices. This overview of the current legal situation represents a snapshot and provides an up-to-date overview of the landscape of European and German medical device law.

## Abstract (de)

Institute und Praxen für Pathologie fungieren als Betreiber, Anwender und Eigenhersteller von *In-vitro*-Diagnostika und unterliegen funktionsabhängig nationalen und europäischen Regularien. Durch das Inkrafttreten der EU-Verordnung über Medizinprodukte (Verordnung (EU) 2017/745, MDR) und der EU-Verordnung über *In-vitro*-Diagnostika (Verordnung (EU) 2017/746, IVDR) ergab sich ein regulatorischer Anpassungsbedarf im deutschen Medizinprodukterecht. Damit wurde ein neuer Rechtsrahmen geschaffen, in dem sich Institute und Praxen für Pathologie je nach ihrer Funktion als Anwender, Betreiber oder Eigenhersteller von *In-vitro*-Diagnostika bewegen. Diese Zusammenstellung der aktuellen Rechtslage stellt eine Momentaufnahme dar und bietet einen aktuellen Überblick über die Landschaft des Medizinprodukterechts.

## Introduction – pathology in the context of medical device law

The introduction of the regulation on in vitro diagnostic medical devices (Regulation (EU) 2017/746, IVDR) and the regulation on medical devices (Regulation (EU) 2017/745, MDR) in the European Union (EU) resulted in the need for regulatory adjustments to German medical device law. This created a new European and national legal framework in which institutes for pathology operate. In this article, we would like to provide an overview of the landscape of medical device law, situate pathology within it and provide practical guidance.

**Figure 1:**
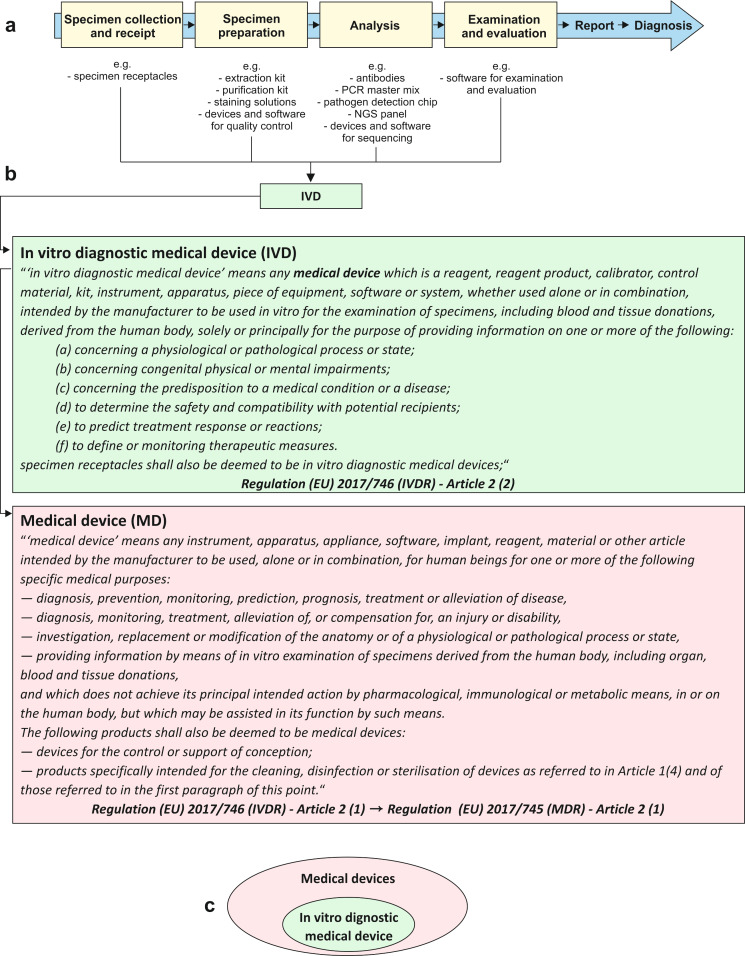
Figure 1 a: In pathology, process chains in analytics for which physicians are responsible lead to valid findings and thus to a diagnosis and optimal patient care. b: Definitions of “in vitro diagnostic medical device” and “medical device” according to the two regulations (EU) 2017/745 (MDR) [18] and 2017/746 (IVDR) [17] c: According to the definitions of the IVDR and MDR, in vitro diagnostic medical devices are a subset of medical devices.

In pathology, process chains under the responsibility of physicians lead to valid findings that enable a diagnosis and the best possible patient care (Figure 1a (Fig. 1)). At each link in the process chain, different types of in vitro diagnostic medical devices (IVDs) can be used individually or in combination with each other. According to the definitions within the two regulations (EU) 2017/746 (IVDR) and (EU) 2017/745 (MDR), *in vitro diagnostic medical devices* are a subset of *medical devices* and are therefore subject to European and German medical device legislation (Figure 1b + c (Fig. 1), see Table 1 (Tab. 1) for definitions) [17], [18]. Products and devices for general laboratory use for which the manufacturer does not declare a specific medical purpose are excluded from this definition and must be considered separately [26]. Nevertheless, these are also an indispensable component of the diagnostic process chain.

From a legal perspective, pathology institutes act as operators and users of in vitro diagnostic medical devices (see Table 1 (Tab. 1) for a definition of “operator” and “user”). The affixing of a CE marking by the manufacturer on an IVD signifies that the manufacturer has declared conformity with the applicable European law. Depending on the risk class of the IVD device, the manufacturer may only add the CE marking after successful assessment by a private conformity assessment body (so-called “Notified Body”). CE marking is a prerequisite for placing the device on the European market. If pathology institutes use IVDs for diagnostic purposes that do not bear this CE marking (e.g. Research Use Only (RUO) devices or self-developed devices) or use IVDR-compliant IVDs (CE-IVDs) outside their intended purpose as specified by the manufacturer or in deviation from their instructions for use, they themselves become in-house manufacturers of so-called in-house in vitro diagnostic medical devices (IH-IVDs) and assume some manufacturer obligations in accordance with Article 5 (5) of the IVDR [25], [26], [30], [31] (see Table 1 (Tab. 1) for a definition of “manufacturer”). This approach is explicitly allowed and desired by the legislator. 

Recital 29 of the preamble to the IVDR emphasizes the particular importance of health institutions and the IVDs they develop themselves. By implementing this recital in Article 5 (5) of the IVDR, the EU legislator recognises the benefits and necessity of IH-IVDs and enables their use for optimal patient care [26] (see also IVDR fact sheet in Table 2 (Tab. 2)). 

Pathology institutes can therefore act multifunctionally as operators and/or users, but also as manufacturers of in-house IVDs. European and German medical device legislation provides a legal framework within which pathology institutes and their employees operate, depending on their respective function. 

## Adjustments to medical device law due to the introduction of the MDR and IVDR

**Figure 2:**
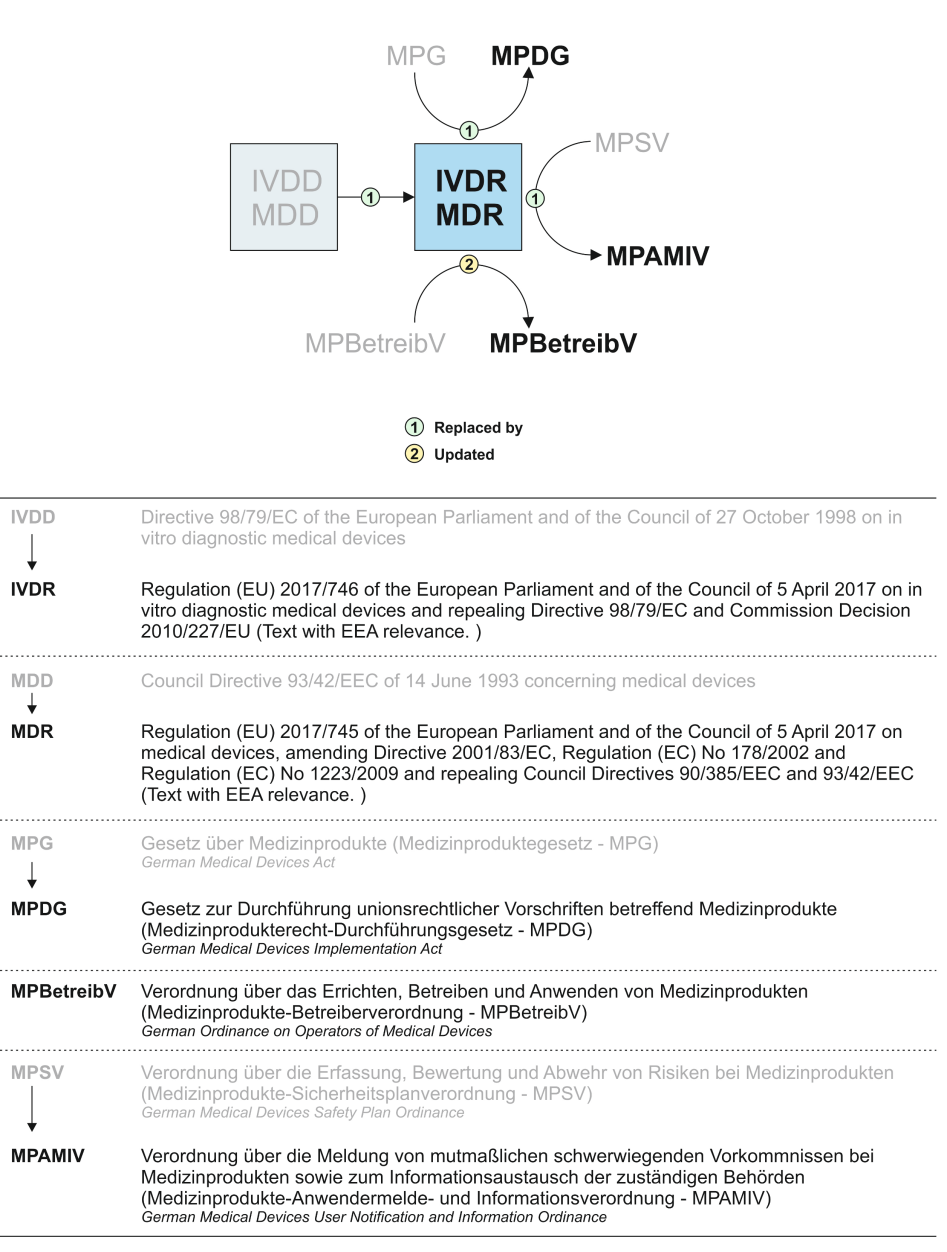
The entry into force of the Regulation (EU) 2017/745 (MDR) and the Regulation (EU) 2017/746 (IVDR) resulted in a need for regulatory adjustments to German medical device law. The changes relevant to pathology institutes for the operation, use and in-house manufacture of in vitro diagnostic medical devices are presented here.

The introduction of the two EU regulations IVDR and MDR is intended to create harmonised framework conditions throughout the Union in order to ensure a high level of health protection for patients and users in all member states. At the same time, a smoothly functioning internal market for medical devices and in vitro diagnostic medical devices is to be ensured (for fact sheets on IVDR and MDR, see Table 2 (Tab. 2) and Table 3 (Tab. 3)) [25]. EU regulations are binding legal acts that apply directly and uniformly in all Union member states as soon as they come into force, without having to be transposed into national law. Nevertheless, extensive adjustments are necessary and also permitted in national medical device law, for example to define responsibilities, inspection activities, penalties and national requirements, which must not, however, contradict the EU regulations (Figure 2 (Fig. 2)).

In Germany, the adaptation of national medical device law to the new EU regulations is implemented by an adaptation act, called “Medizinprodukte-EU-Anpassungsgesetz” (MPEUAnpG) [21]. This includes the German Medical Devices Implementation Act “Medizinprodukterecht-Durchführungsgesetz” (MPDG, Article 1 in the MPEUAnpG), which replaces the former Medical Devices Act “Medizinproduktegesetz” (MPG) (Figure 2 (Fig. 2)). In contrast to the replaced MPG, the new MPDG is an accompanying act that supplements the two EU regulations MDR and IVDR with national requirements.

### MPDG – Medizinprodukterecht-Durchführungsgesetz: German Medical Devices Implementation Act

**Figure 3:**
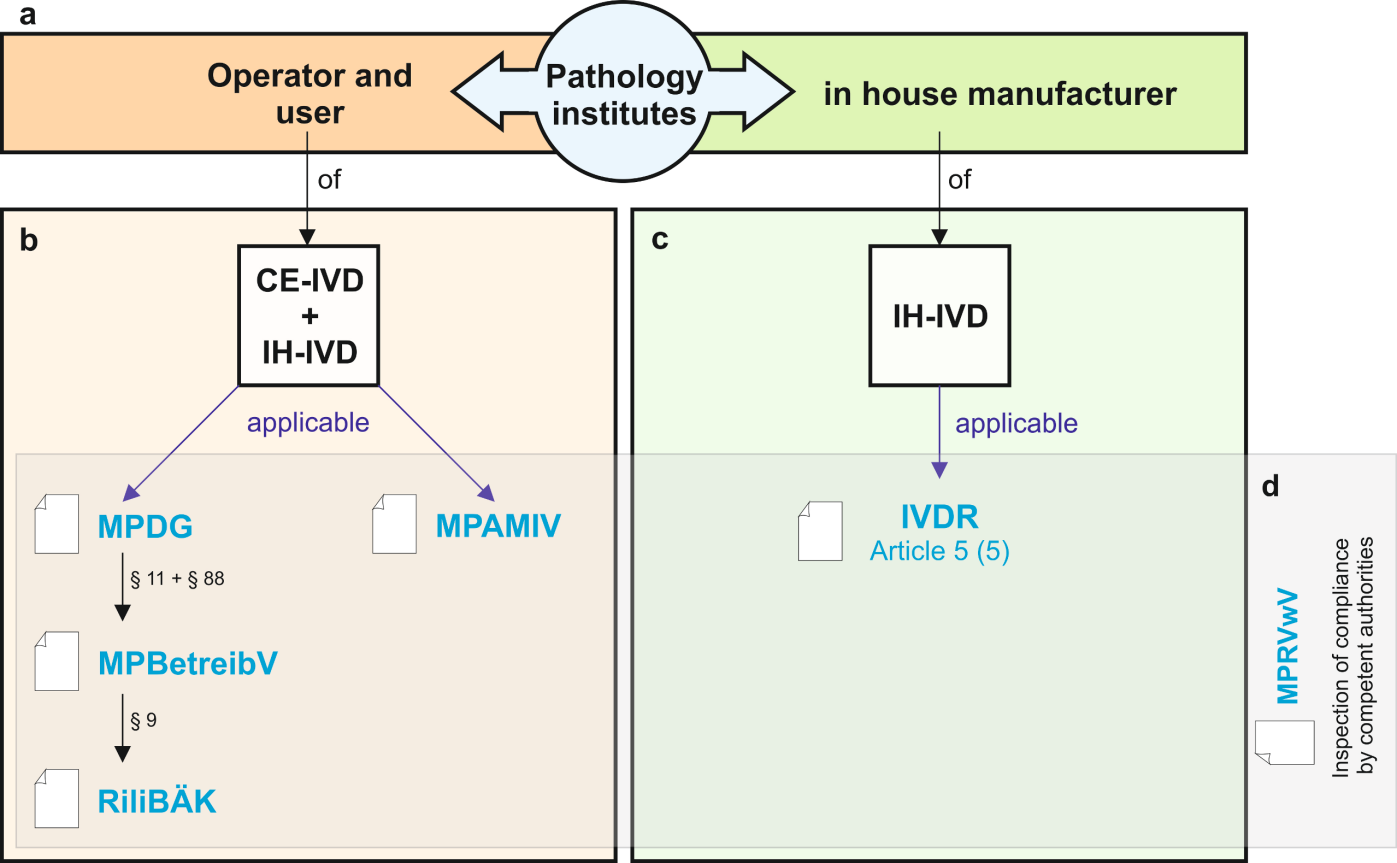
Figure 3 a: Institutes of pathology act as operators, users and in-house manufacturers of in vitro diagnostic medical devices and must fulfil the respective legal requirements depending on their function. b: Legal framework as an operator and user of in vitro diagnostic medical devices c: Legal framework as an in-house manufacturer of in vitro diagnostic medical devices d: Inspection of compliance with medical device law by competent authorities in accordance with the general administrative regulation MPRVwV

The German Medical Devices Implementation Act “Medizinprodukterecht-Durchführungsgesetz” (MPDG) [22] adapts the national medical device law to the new EU regulations IVDR and MDR and replaces the former Medical Devices Act “Medizinproduktegesetz” (MPG) (Figure 2 (Fig. 2), for fact sheet on MPDG see Table 4 (Tab. 4)). The MPDG also applies to devices within the scope of the IVDR and therefore also applies to pathology institutes, which can act as in-house manufacturers, operators and users of IVDs (Figure 3 (Fig. 3)). The MPDG regulates the penalties and fines for non-compliance with the IVDR and the MPDG itself. Institutes for pathology are subject to general market surveillance by the competent regulatory authorities (chapter 5, § 77). They may only operate and use IVDs that are free of defects so that patients, users and potential third parties are not endangered (§ 11, sentence 1). In addition, they must comply with the German Ordinance on Operators of Medical Devices “Medizinprodukte-Betreiberverordnung” (MPBetreibV, § 11, sentence 2). The responsibilities for pathology institutes as operators and users of IVDs are therefore primarily defined in the MPBetreibV. 

### MPBetreibV – die Medizinprodukte-Betreiberverordnung: German Ordinance on Operators of Medical Devices

The German Ordinance on Operators of Medical Devices “Medizinprodukte-Betreiberverordnung” (MPBetreibV) has been a national regulation to the former German Medical Devices Act “Medizinproduktegesetz” (MPG) since 1998. Since the MPG was replaced by the MPDG on the basis of the MPEUAnpG, the MPBetreibV was also revised, most recently in April 2021 [32]. Currently, a draft from the German Federal Ministry of Health (Bundesgesundheitsministerium) provides for a possible amendment to the MPBetreibV [10]. 

The ordinance MPBetreibV implements § 11 of the MPDG in conjunction with § 88, sentence 1 no. 6 a), b) and c). It applies to the operation and use of medical devices and in vitro diagnostic medical devices, including the associated activities, such as set-up, maintenance measures and regular inspections, and describes the applicable responsibilities for operators and users (for fact sheet on MPBetreibV see Table 5 (Tab. 5)).

MPBetreibV specifies how institutions that carry out laboratory medical tests must act as operators of IVDs in order to ensure safe and proper use. To achieve this, the MPBetreibV specifies both operator and user obligations that are relevant for pathology institutes and their staff. The aim of this ordinance is to ensure the safe and proper use of medical devices within the health institution. Medical devices, and therefore also IVDs, may only be used in accordance with their intended purpose and only by competent and appropriately trained personnel (§§ 4 and 5). However, the IVDR indicates an exception to this – the possible use of devices in deviation from the manufacturer’s intended purpose. Health institutions may deviate from the manufacturer’s intended purpose in order to meet the specific needs of target patient groups, as long as they fulfil all the conditions specified in Article 5 (5) of the IVDR. Therefore, they have to re-define and document a new intended purpose. For this, they have to take on some of the manufacturer obligations regulated in the IVDR, e.g. by ensuring conformity with the general safety and performance requirements set out in Annex I of the IVDR (Article 5 (5), sentence 1) [24], [25]. In order to achieve greater coherence between the MPBetreibV and the IVDR, the German Federal Ministry of Health’s draft proposal to amend the MPBetreibV provides for the repeal of Section 4, paragraph 1, so that the use of medical devices in accordance with their intended purpose will no longer be required under the revised MPBetreibV [10]. 

Irrespective of this, all IVDs must be used only if their functionality and absence of defects can be guaranteed before use (§ 4). This can be achieved by checking expiry dates and maintenance measures before use, as well as by carrying out functional checks. Maintenance measures, such as inspections and servicing, and functional tests must be carried out regularly and continuously and in accordance with the manufacturer’s instructions.

In accordance with the stipulations of § 6, health institutions with a minimum of 20 employees are obliged to appoint a medical device safety officer who can be contacted via a functional email address on the website as a central point of contact within the health institution, e.g. for authorities or for reporting risks and incidents (§ 6). Additionally, the medical device safety officer is responsible for coordinating internal processes required to fulfil the reporting obligation in accordance with the German Medical Devices User Notification and Information Ordinance “Medizinprodukte-Anwendermelde- und Informationsverordnung” (MPAMIV, see below).

The MPBetreibV stipulates that facilities that carry out laboratory medical tests must implement a quality assurance system to assure the required quality, safety and performance in the use of in vitro diagnostic medical devices and to ensure the reliability of the results obtained (§ 9 (1), sentence 1). An orderly quality assurance system is assumed if the laboratory complies with Part A of the guideline of the German Medical Association (Bundesärztekammer) for quality assurance of laboratory medical tests “Richtlinie der Bundesärztekammer zur Qualitätssicherung laboratoriumsmedizinischer Untersuchungen” (RiliBÄK, § 9 (1), sentence 2). 

### RiliBÄK-Guideline – Richtlinie der Bundesärztekammer zur Qualitätssicherung laboratoriumsmedizinischer Untersuchungen: Guideline of the German Medical Association (Bundesärztekammer) for Quality Assurance of Laboratory Medical Tests

The application of the guideline of the German Medical Association (Bundesärztekammer) for quality assurance of laboratory medical tests (Richtlinie der Bundesärztekammer zur Qualitätssicherung laboratoriums-medizinischer Untersuchungen, RiliBÄK) has been stipulated in the MPBetreibV since 2001. The current version of the RiliBÄK was published in the German Medical Journal (Deutsches Ärzteblatt) on 30 May 2023 [5]. As a Medical Association guideline, it is neither a law nor a legal regulation, but physicians in Germany are obliged to participate in the quality assurance measures introduced by the German Medical Association (Bundesärztekammer) in accordance with § 5 of the Medical Association’s professional code of conduct (Berufsordnung) (MBO-Ä 1997) [4]. Compliance with the RiliBÄK-guideline is also recommended due to the presumption of conformity in § 9 of the MPBetreibV: its application fulfils the requirement of the MPBetreibV for a suitable quality assurance system when operating and using IVDs. Any deviating action requires special justification [4].

The aim of the RiliBÄK-guideline is to ensure and continuously improve the quality of laboratory medical tests and to minimise risks for patients and users (see Table 6 (Tab. 6) for fact sheet on RiliBÄK-guideline) [7]. This objective is to be achieved by defining fundamental requirements for the structural and process quality of laboratory medical tests in healthcare, for the necessary quality management and for permanent quality assurance. Quality assurance should be risk-based. The test-specific risk assessment must be documented. 

RiliBÄK-guideline contains two parts relevant for laboratory medical tests, A and B. Part A specifies the basic requirements for the quality assurance of analyses (e.g. mandatory participation in external round robin tests (Part A, Chapter 8.2)). Part A describes requirements for the pre-analysis (sample submission), for the analysis itself, up to the post-analysis (Part A, Chapter 6). It is stipulated that only validated analysis methods must be used. The validation and the results obtained must be documented, as well as the analysis procedure, which then must be available at the workplaces. 

Part B specifies the test-specific requirements for the quality of results and the relevant principles of quality assurance (e.g. number of round robin tests per calendar year depending on the analysis). Part B is divided into five special subparts B1 to B5:

- B1: Quantitative laboratory medical tests
- B2: Qualitative laboratory medical tests
- B3: Direct detection and characterisation of pathogens
- B4: Ejaculate analyses
- B5: Molecular genetics and cytogenetic analyses

Subparts B1, B2, B3 and B5 would primarily apply to the field of pathology. Part B5 was completely rewritten in May 2023 when the RiliBÄK-guideline was updated due to technical progress. The German Medical Association provides a “Frequently Asked Questions Sheet” for RiliBÄK-guideline on its website [6]. According to a recent decision by the German Medical Association, the concept of a possible extension of the RiliBÄK-guideline to include the field of pathology is to be developed so that all morphology-based procedures (e.g. immunohistochemistry) would be covered by this guideline [11]. 

### MPAMIV – Medizinprodukte-Anwendermelde- und Informationsverordnung: German Medical Devices User Notification and Information Ordinance

The introduction of the EU-regulations IVDR and MDR, along with the replacement of the German Medical Devices Act “Medizinproduktegesetz” (MPG) by the German Medical Devices Implementation Act “Medizinprodukterecht-Durchführungsgesetz” (MPDG), resulted in the replacement of the German Medical Devices Safety Plan Ordinance “Medizinprodukte-Sicherheitsplanverordnung” (MPSV) by the new German Medical Devices User Notification and Information Ordinance “Medizinprodukte-Anwendermelde- und Informationsverordnung” (MPAMIV) on 26 May 2021 [33] (Figure 2 (Fig. 2)). 

The ordinance MPAMIV regulates the notification and information exchange procedure for *suspected serious incidents* involving IVDs (see Table 1 (Tab. 1) for definition). The MPAMIV is structured into two sections (see Table 7 (Tab. 7) for fact sheet on MPAMIV). Section 1, which includes the scope and notification procedure, addresses the reporting of suspected serious incidents and is relevant for pathology institutes acting as operators and users of IVDs. Section 2 of the MPAMIV defines the exchange of information between the competent authorities. 

The ordinance MPAMIV fulfils the mandate set out in Article 82 (10) of the IVDR for EU member states to regulate the notification procedure and obligation for operators and users of IVDs. Suspected serious incidents in connection with IVDs made available on the EU market (CE-IVDs) must be reported by the operator or user of IVDs to the competent federal authority (§ 3). This also applies to physicians who become aware of such incidents in the course of their medical practice. Patients can also make notifications directly, but unlike operators and users, they are not obliged to do so. The notification is made via the German Medical Devices Information and Database System (Deutsche Medizinprodukteinformations- und Datenbanksystem, DMIDS) in accordance with § 86 of the German Medical Devices Implementation Act (MPDG) to the federal authority, either to the Federal Institute for Drugs and Medical Devices (Bundesinstitut für Arzneimittel und Medizinprodukte – BfArM, [8]) or to the Federal Institute for Vaccines and Biomedicines (Paul-Ehrlich-Institut – PEI, [27]). The coordination of the necessary processes can be carried out via the authorised medical device safety officer (according to MPBetreibV, § 6). An information sheet on the notification obligation of the German Federal Council (Regierungspräsidium) of Baden-Württemberg recommends that each institute should have a procedural instruction for the notification process that contains the responsibilities, the reporting deadline, the web addresses of the reporting forms and the regulations for storing the affected device [28]. 

Notification of suspected serious incidents is only mandatory for medical devices that are made available on the European Union market (CE-IVD) (IVDR, Article 82 (1)). Devices that are manufactured in-house by health institutions in accordance with IVDR, Article 5 (5), and are used exclusively within the health institution (IH-IVD) are therefore presumably not affected by this reporting obligation, as according to IVDR, Article 5 (5), they may not be made available on the market (see Table 1 (Tab. 1) for definition). 

## Supervisory measures for medical device law

Institutes of pathology act as operators, users and in-house manufacturers of in vitro diagnostic medical devices and must fulfil the respective legal requirements depending on their function (Figure 3a (Fig. 3)). European and German medical device legislation provide a legal framework within which pathology institutes and their employees operate, depending on their function as described above (Figure 3b + c (Fig. 3)). According to Article 5 (5) of the IVDR, the competent authorities of the EU member states shall be permitted access to inspect the activities of the health institutions. In Germany, the details of this authorization for inspection are regulated in § 79, section 1, no. 1 and 2 of the MPDG. According to § 77 of the MPDG, the federal states are responsible for monitoring compliance with medical device law. In order to ensure a uniform and nationwide approach to quality-assured and risk-based inspection by the competent authorities of the federal states, the General Administrative Regulation on the Implementation of Medical Devices Law “Medizinprodukterecht-Durchführungsvorschrift” (MPRVwV) was issued on 12 June 2023 on the basis of § 89 MPDG (Figure 3d (Fig. 3)) [9].

### MPRVwV – Medizinprodukterecht-Durchführungsvorschrift: German General Administrative Regulation on the Implementation of Medical Devices Law

The German General Administrative Regulation on the Implementation of Medical Devices Law “Medizinprodukterecht-Durchführungsvorschrift” (MPRVwV) issued in June 2023 is aimed at the authorities of the federal states that are responsible for implementing medical device law in order to ensure a uniform approach throughout Germany [9]. This general administrative regulation stipulates that the monitoring measures and inspection intervals must be carried out on the basis of annual plans in a risk-based and quality-assured manner (see Table 8 (Tab. 8) for fact sheet on MPRVwV). For this purpose, the competent authorities of the federal states should define principles and requirements in accordance with § 3 (Principles of inspection) and § 5 (Quality assurance system). At least one qualified person must be assigned to the authority to carry out the quality assurance measures. Inspections are generally to be carried out on site and are performed by checking device-related documents and information, by physical inspections or by laboratory tests. Such inspections may be conducted on an annual basis, or on an ad hoc basis. They may be carried out both announced and unannounced. In addition, they should consider the special features of the activities in laboratories and facilities (§ 8). 

## Further guidelines and standards

As healthcare providers, pathology institutes in Germany are legally obliged to implement and continuously improve a quality management system (QMS) (Sozialgesetzbuch (SGB V), Section 9 “Sicherung der Qualität der Leistungserbringung” [29]). The quality management guideline (Qualitätsmanagement-Richtlinie (QM-RL)) of the Federal Joint Committee (Gemeinsamer Bundesausschuss (G-BA)) describes the requirements for the implementation and realisation of quality management for medical institutions that work for people with statutory health insurance (SHI) [19]. Part A of the guideline QM-RL contains the framework provisions that apply to all sectors (e.g. risk, complaint and error management), as well as the sector-specific specifications, e.g. for SHI-accredited medical care (Part B).

The German Ordinance on Operators of Medical Devices (MPBetreibV) requires the implementation of a quality assurance system in accordance with the state of the art in medical science and technology to maintain the necessary quality, safety and performance in the use of IVDs and to ensure the reliability of the results obtained (§ 9). The IVDR also requires an appropriate quality management system if the institution acts as a manufacturer of IH-IVDs (Article 5 (5), b) [17]). The IVDR further requires in Article 5 (5), c): *“the laboratory of the health institution is compliant with standard EN ISO 15189 or where applicable national provisions, including national provisions regarding accreditation”* [17]. Standards represent the state of the art and define standardised and harmonised requirements, e.g. for processes, devices or management systems. However, they are not legally binding unless compliance with them is specifically prescribed by law. The standard EN ISO 15189 explicitly mentioned here describes the requirements for the quality and competence of medical laboratories and was revised in terms of structure and content in 2022 and has been available as the German version DIN EN ISO 15189:2023-03 since 2023. However, as this standard does not contain any requirements for the (in-house) manufacture of devices, it does not fully comply with Article 5 (5), b), which also stipulates a quality management system for manufacturing. 

**Figure 4:**
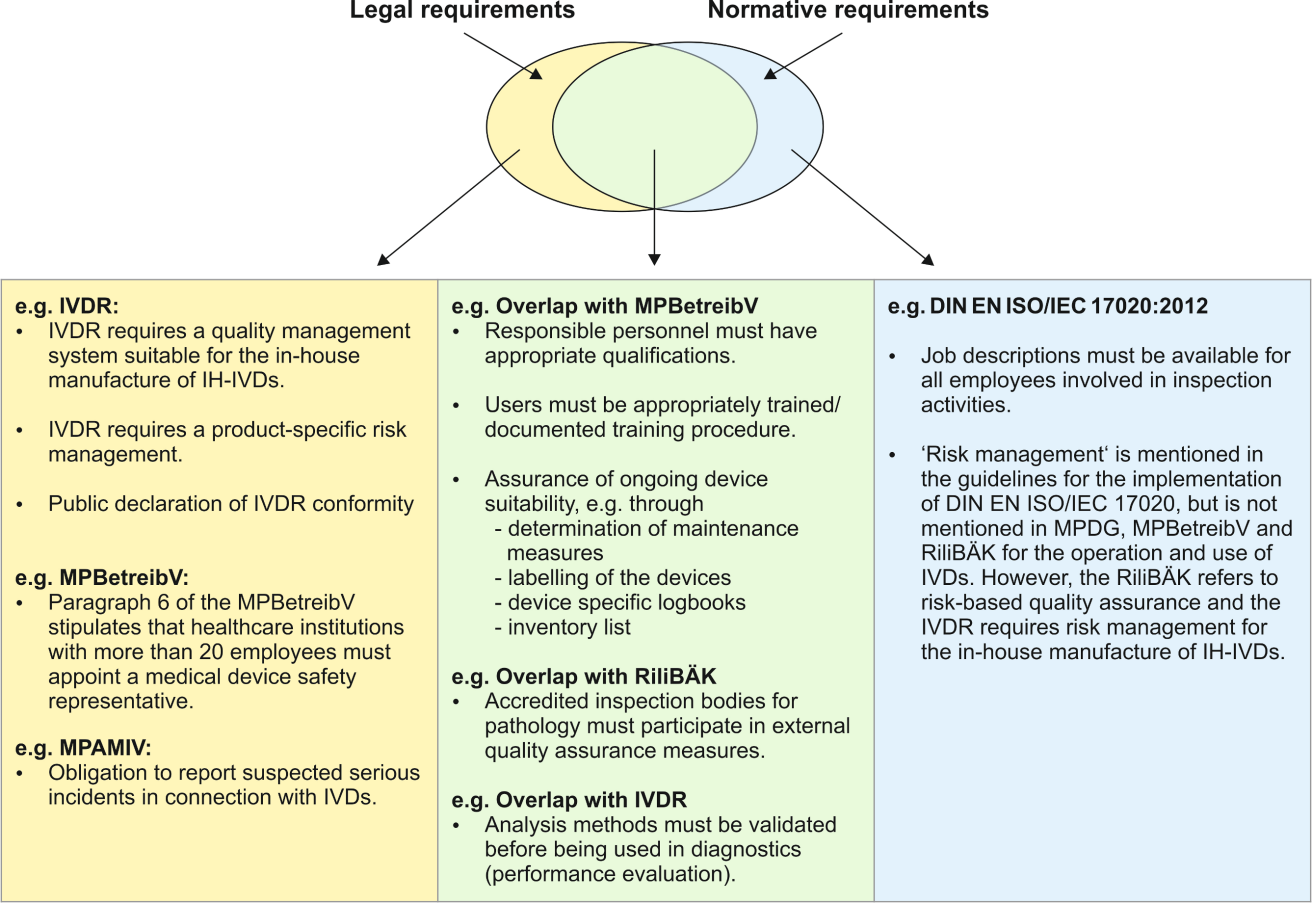
Accreditation in the fields of pathology, neuropathology and dermatohistology is based on the standard DIN EN ISO/IEC 17020 (blue). The standard covers a large part of the legal requirements (green). However, conformity with the standard or accreditation itself does not cover all legal requirements (yellow).

The German Accreditation Body DAkkS (Deutsche Akkreditierungsstelle GmbH) has the legal mandate as the national accreditation authority of the Federal Republic of Germany to carry out the voluntary accreditation of conformity assessment bodies upon request (Regulation (EC) No 765/2008 [16], German Act for Accreditation Bodies (Akkreditierungsstellengesetzes (AkkStelleG)) [20]). With an accreditation, the DAkkS confirms that the audited organisation can perform its work competently “in accordance with the requirements of internationally valid standards, legal principles and relevant regulation” [15]. Accreditation is intended to serve the public interest, but is not mandatory for legal compliance for health institutions. Accreditation by itself does not guarantee legal compliance (Figure 4 (Fig. 4)). The accreditation of medical laboratories in Germany is based on the standard DIN EN ISO 15189, supplemented by the RiliBÄK-guideline [12].

In Germany, over 100 institutes for pathology, neuropathology and dermatohistology are accredited as inspection bodies by the DAkkS in accordance with the standard DIN EN ISO/IEC 17020 (see search result in [14] with the following filter: inspection body, pathology, active; hits: 110), [23]. The focus of accreditation according to DIN EN ISO/IEC 17020 is the expert assessment of the physician on the diagnosis and thus the confirmation of the professional competence of the inspection body [12]. The requirements of the standard DIN EN ISO 15189 must also be taken into account for successful accreditation and the DAkkS confirms conformity with the requirements of this standard for all examination methods within the scope of accreditation in the annex to the certificate. 

The catalogue of requirements of the former pathology/neuropathology sector committee (identifier: 71 SD 4 001 [13]) provided interpretations and recommendations for the standard DIN EN ISO/IEC 17020 and referred to the legal basis in its requirement for chapter 7.1.4 of the standard DIN EN ISO/IEC 17020. Although the sector committees and their assistance are currently being transferred to a new established Expert Council and new checklists due to reorganisation within the DAkkS, it makes sense to always have an up-to-date overview of the legal situation, including medical device law, even if the standard itself does not explicitly require this.

## Discussion and conclusion

In the performance of diagnostic examination procedures in pathology under the responsibility of a physician, medical devices in the form of in vitro diagnostic medical devices are used as an integral part of the diagnostic process. In all functions, whether as an operator, user or in-house manufacturer of IVDs, patient and user safety and valid diagnosis are always at the centre of pathology’s focus. In order to ensure a high level of safety and health protection, there are legal and normative principles at national and European level that form a legal framework and the state of the art for pathology institutes. 

The association of the scientific medical societies in Germany (Arbeitsgemeinschaft der Wissenschaftlichen Medizinischen Fachgesellschaften e.V. (AWMF)) regularly publishes statements on (planned) changes to medical device law. Thus, the association presents and comments on the requirements that health institutions must fulfil for the in-house manufacture of IH-IVDs due to the IVDR and points out definitional gaps in the regulation [1]. The AWMF also commented on the current draft bill of the German Federal Ministry of Health (Bundesgesundheitsministeriums) for the planned amendment of the German Ordinance on Operators of Medical Devices (MPBetreibV) [3]. This draft provides for an expansion of its scope of application, which is due to increasing digitalisation. It is now also intended to regulate the handling of medical device software. This is in line with the new inclusion of “software” in the definitions of “medical device” and “in vitro diagnostic medical device” in the MDR and IVDR (Table 1 (Tab. 1)). In the previous medical device directive (MDD) and in vitro diagnostic medical device directive (IVDD) software was not yet part of the definitions. Furthermore, it is planned to delete § 4, section 1 of the ordinance MPBetreibV. This section currently permits the use of medical devices and IVDs only in accordance with their intended purpose as specified by the manufacturer, whereas the IVDR also permits the use outside of the intended purpose and thus enables health institutions to address the specific needs of target patient groups (Article 5 (5)). The planned repeal of this section of the MPBetreibV thus underlines the freedom of method choice of pathologists and represents harmonisation with the IVDR.

In vitro diagnostic medical devices are physical devices that are used within a process chain in analytics under professional medical responsibility to enable a profound diagnosis. It is important to make a clear distinction between the IVD as a physical medical device from the professional performance of the diagnostic procedure as a process and as medical service under medical responsibility [34], [35]. This is currently only partially done in the guidance document MDCG-2023-1 of the Medical Device Coordination Group (MDCG) for health institutions [26]. The MDCG and its tasks are described in Articles 98 and 99 of the IVDR. It has the task of developing guidelines to ensure effective and harmonised implementation of the IVDR, but these are not legally binding. Guideline MDCG-2023-1 is intended to provide recommendations and assistance to health institutions manufacturing IH-IVDs by summarising and commenting on the requirements of Article 5 (5). The PCR master mix as a component is mentioned here as an example of an IVD, but not PCR-method as a test procedure. The AWMF Ad-hoc Commission on In Vitro Diagnostic Medical Devices points out that the performance of diagnostic procedures as a process and under medical responsibility is not an IVD, but instead a medical service that is not regulated by the IVDR. Its quality assurance is subject to medical self-administration in Germany, and therefore calls for a precise demarcation [2]. 

## Conclusion for practice

- As operators and users of CE-IVDs and as manufacturers, operators and users of IH-IVDs, institutes for pathology are subject to European and national medical device law (see Figure 3a + b (Fig. 3) for the current legal situation in Germany).
- The introduction of the two EU regulations IVDR and MDR resulted in a regulatory adjustment requirement in German medical device law (see Figure 2 (Fig. 2)).
- Pathology institutes can be inspected for compliance with the medical device law. In Germany, the inspections are carried out by the competent authorities in the individual federal states (see Figure 3d (Fig. 3)).
- Conformity with standards and DAkkS accreditation provide a good basis for compliance with legal requirements, but it does not include all legal requirements for the in-house manufacture (see Figure 4 (Fig. 4)) of IH-IVDs and for the operation and use of CE- and IH-IVDs.

## Notes

### Competing interests

- Andy Kahles: There is no conflict of interest.
- Hannah Goldschmid: There is no conflict of interest.
- Anna-Lena Volckmar: Personal fees from AstraZeneca and Novartis unrelated to the submitted work
- Daniel Kazdal: There is no conflict of interest.
- Ulrich M. Gassner: There is no conflict of interest.
- Peter Schirmacher: There is no conflict of interest.
- Albrecht Stenzinger: Advisory Board/Talk: AGCT, Aignostics, AstraZeneca, Bayer, BMS, Eli Lilly, Illumina, Incyte, Janssen, MSD, Novartis, Pfizer, Roche, Seattle Genetics, Takeda, Thermo Fisher; Grants: Bayer, BMS, Chugai, Incyte
- Karl-Friedrich Bürrig: There is no conflict of interest.
- Vanessa Kääb-Sanyal: There is no conflict of interest.
- Christa Flechtenmacher: There is no conflict of interest.
- Michael Vogeser: There is no conflict of interest.
- Monika Brüggemann: There is no conflict of interest.

## Figures and Tables

**Table 1:**
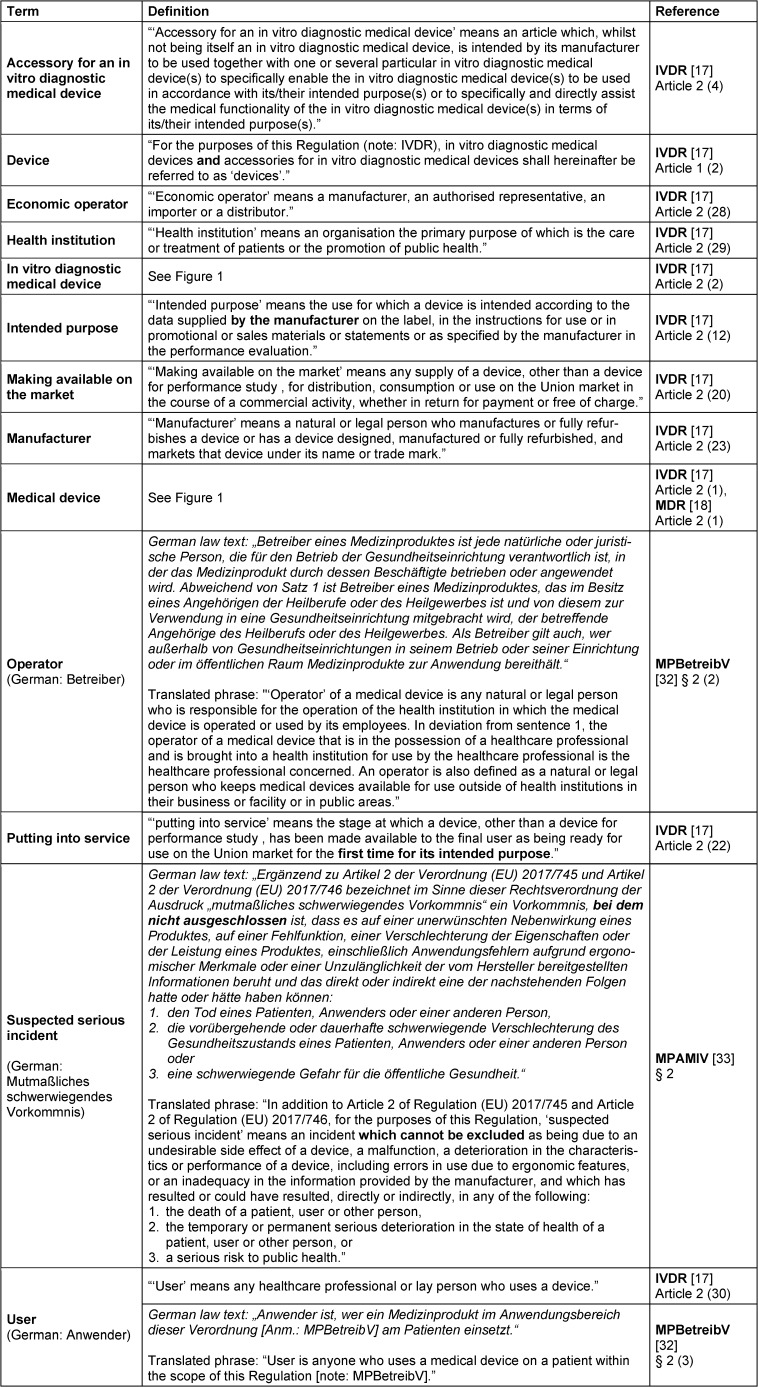
Definitions with the relevant legal bases (sorted alphabetically). Bold type within the definition has been added for highlighting purposes and is not part of the respective original text. Definitions referring to German law texts are given in German. A non-official translation by the authors is added in each case.

**Table 2:**
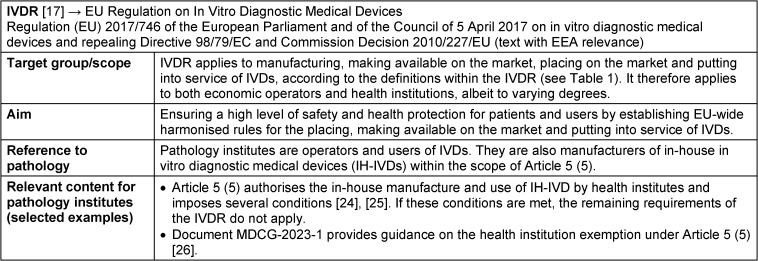
Fact sheet Regulation (EU) 2017/746 IVDR

**Table 3:**
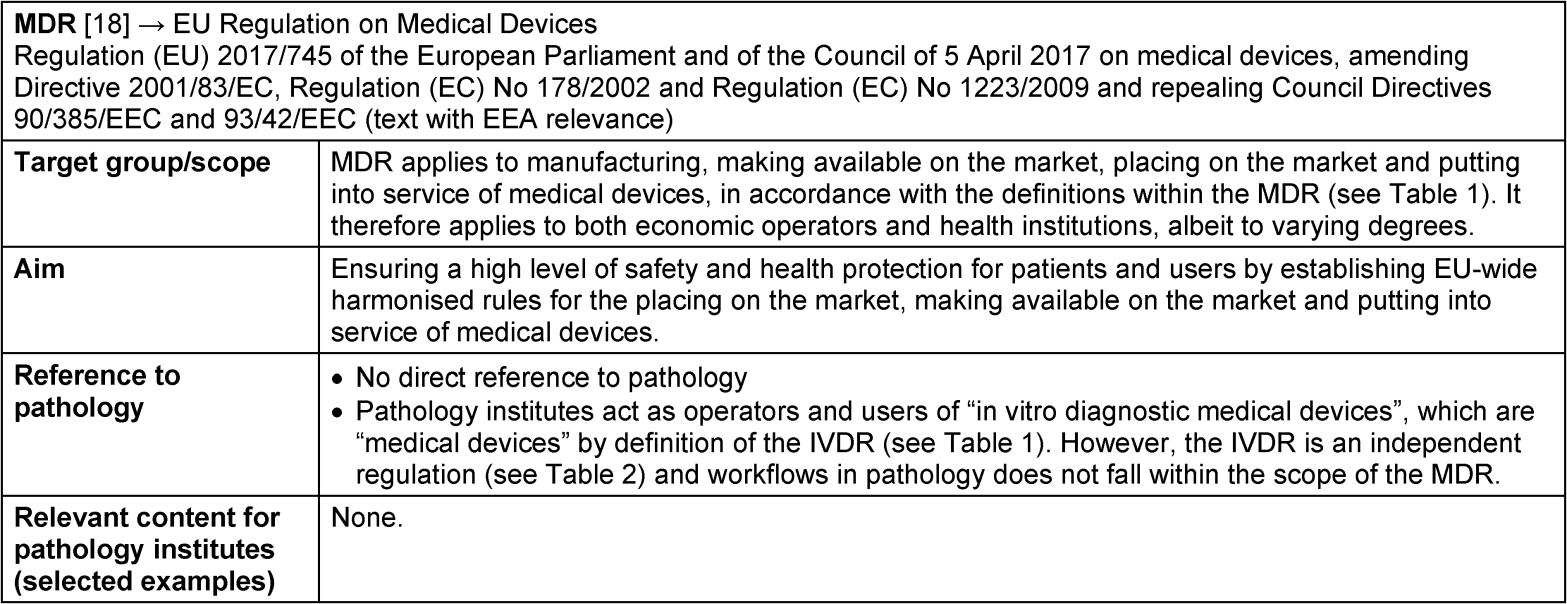
Fact sheet Regulation (EU) 2017/745 MDR

**Table 4:**
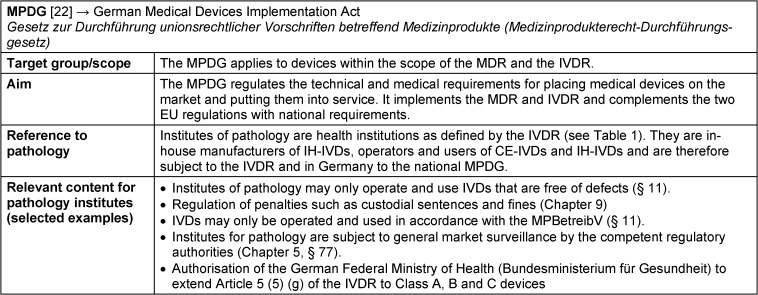
Fact sheet German Medical Devices Implementation Act (MPDG)

**Table 5:**
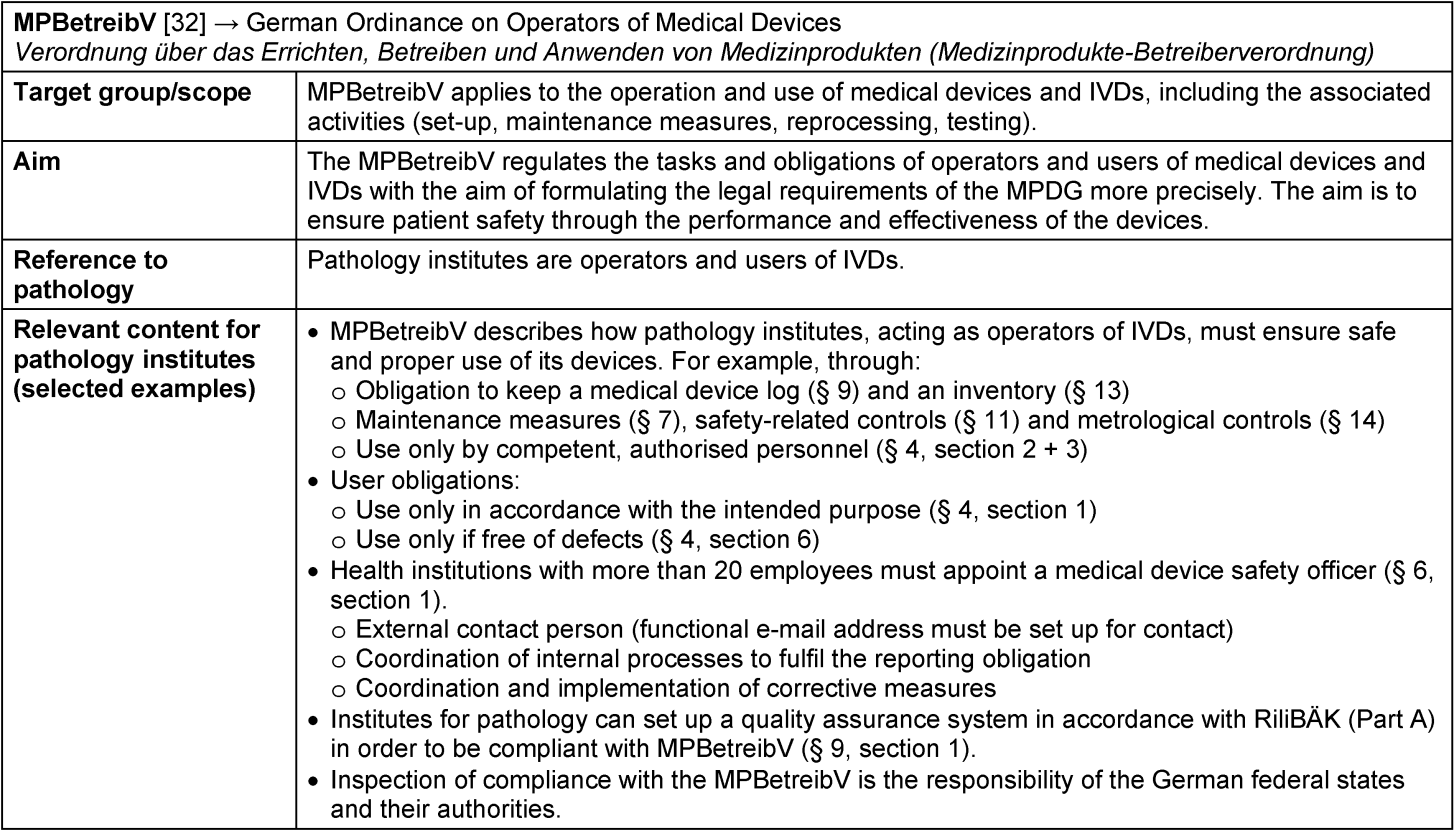
Fact sheet German Ordinance on Operators of Medical Devices (MPBetreibV)

**Table 6:**
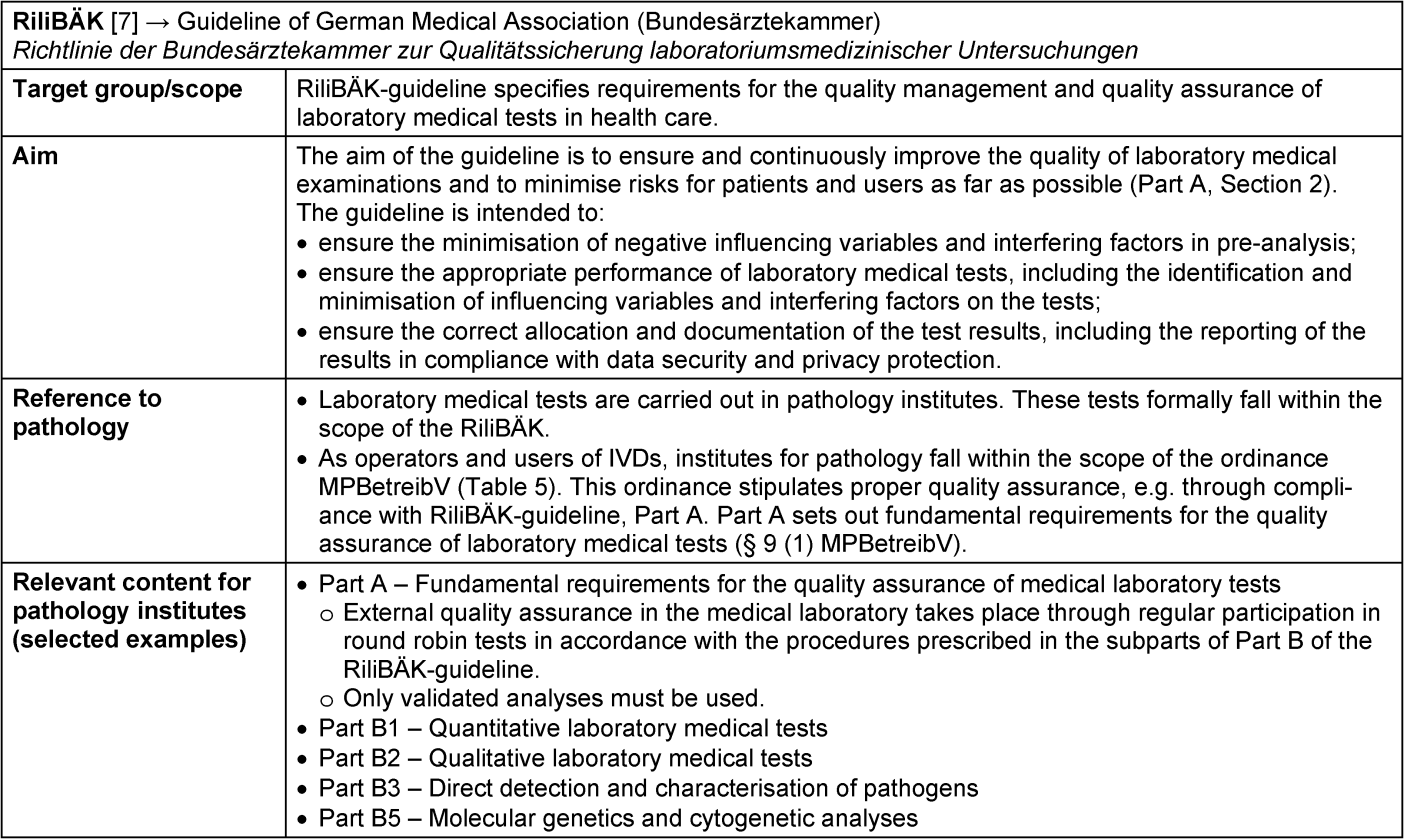
Fact sheet Guideline of German Medical Association for quality assurance of laboratory medical tests (RiliBÄK)

**Table 7:**
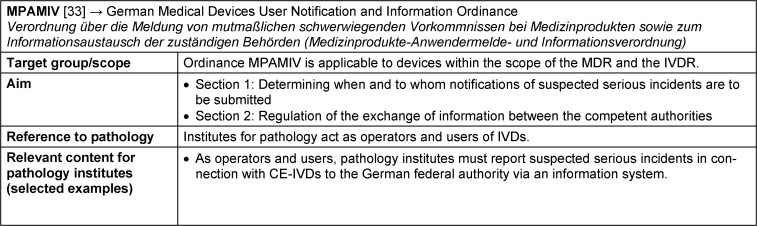
Fact sheet German Medical Devices User Notification and Information Ordinance (MPAMIV)

**Table 8:**
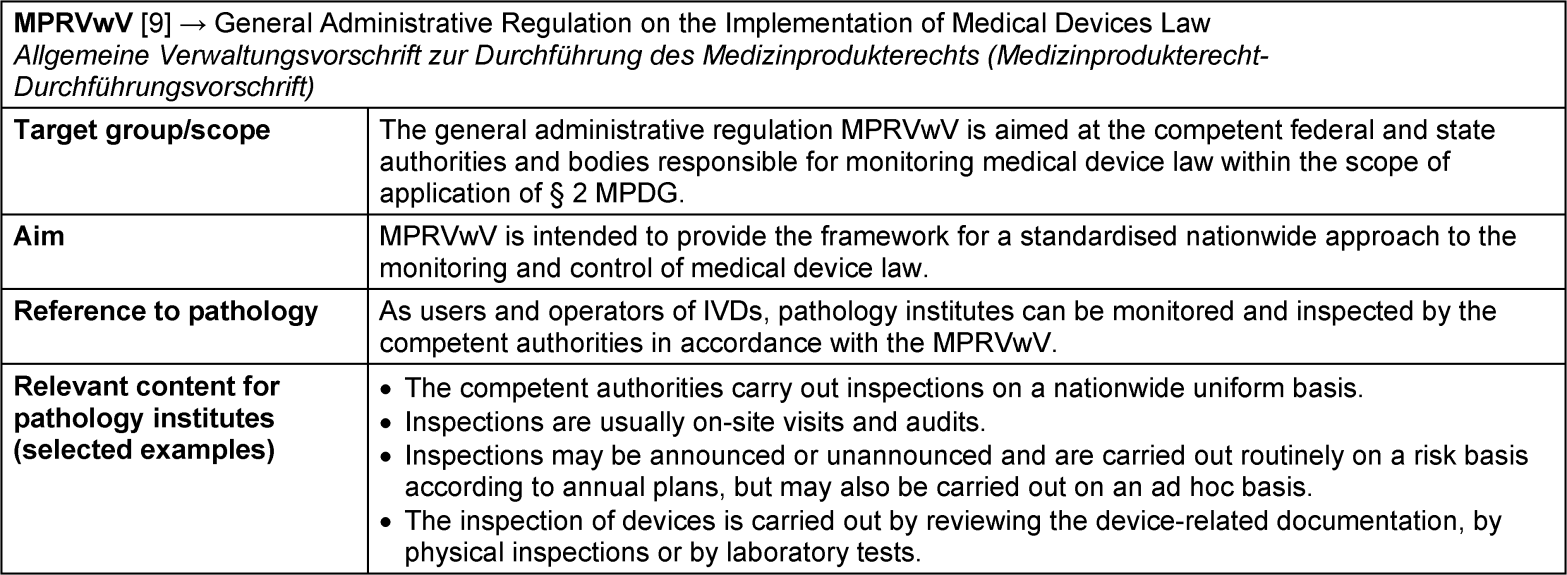
Fact sheet General Administrative Regulation on the Implementation of Medical Devices Law (MPRVwV)
